# Germline Variants in Phosphodiesterase Genes and Genetic Predisposition to Pediatric Adrenocortical Tumors

**DOI:** 10.3390/cancers12020506

**Published:** 2020-02-22

**Authors:** Emilia Modolo Pinto, Fabio R. Faucz, Luana Z. Paza, Gang Wu, Elizabeth S. Fernandes, Jerome Bertherat, Constantine A. Stratakis, Enzo Lalli, Raul C. Ribeiro, Carlos Rodriguez-Galindo, Bonald C. Figueiredo, Gerard P. Zambetti

**Affiliations:** 1 Department of Pathology, St. Jude Children’s Research Hospital, Memphis, TN 38105-2794, USA; 2 Section on Genetics and Endocrinology, Eunice Kennedy Shriver National Institute of Child Health and Human Development (NICHD), National Institutes of Health, Bethesda, MD 20892-1862, USA; fabio.faucz@nih.gov (F.R.F.); stratakc@cc1.nichd.nih.gov (C.A.S.); 3 Pele Pequeno Principe Research Institute and Faculdades Pequeno Principe, Curitiba PR 80250-200, Brazil; lu.paza27@gmail.com; 4 Center for Applied Bioinformatics, St. Jude Children’s Research Hospital, Memphis, TN 38105-2794, USA; gang.wu@stjude.org; 5 Pele Pequeno Principe Research Institute, Faculdades Pequeno Principe, Curitiba PR 80250-200, Brazil; elizabeth.fernandes@pelepequenoprincipe.org.br; 6 Institut Cochin, Université Paris Descartes, Paris. Service d’Endocrinologie, Centre de référence des maladies rares de la surrénale, Assistance Publique Hôpitaux de Paris, Hôpital Cochin, F-75014 Paris, France; jerome.bertherat@aphp.fr; 7 Institut de Pharmacologie Moléculaire et Cellulaire CNRS, 06560 Valbonne, France; lalli@ipmc.cnrs.fr; 8 Department of Oncology, St. Jude Children’s Research Hospital, Memphis, TN 38105-2794, USA; raul.ribeiro@stjude.org; 9 Department of Global Pediatric Medicine, St. Jude Children’s Research Hospital, Memphis, TN 38105-2794, USA; carlos.rodriguez-galindo@stjude.org; 10 Pele Pequeno Principe Research Institute, Faculdades Pequeno Principe, Centro de Genética Molecular e Pesquisa do Câncer em Crianças (CEGEMPAC) and Departamento de Saúde Coletiva, Federal University of Paraná, Curitiba PR 80250-200, Brazil; bonald@ufpr.br

**Keywords:** phosphodiesterase, cAMP pathway, adrenocortical tumor, *TP53*, 11p

## Abstract

Phosphodiesterases (PDEs) form a superfamily of enzymes that catalyze the hydrolysis of cyclic nucleotides adenosine 3′5′-cyclic monophosphate (cAMP) and guanosine 3′5′-cyclic monophosphate (cGMP) to their inactive 5′ monophosphates. cAMP plays a critical role as a second messenger in endocrine tissues, and activation of cAMP signaling has been reported in endocrine tumors. Germline variants in PDEs have been associated with benign cortisol-secreting adrenocortical adenomas and testicular germ cell cancer but not adrenocortical carcinoma. We performed whole genome sequencing (WGS) and whole exome sequencing (WES) of paired blood and tumor samples from 37 pediatric adrenocortical tumors (ACTs). Germline inactivating variants in PDEs were observed in 9 of 37 (24%) patients. Tumor DNA analysis revealed loss of heterozygosity, with maintenance of the mutated allele in all cases. Our results suggest that germline variants in PDEs and other regulators of the cAMP-signaling pathway may contribute to pediatric adrenocortical tumorigenesis, perhaps by cooperating with germline hypomorphic mutant *TP53* alleles and uniparental disomy of chromosome 11p15 (Beckwith–Wiedemann syndrome).

## 1. Introduction

Cyclic nucleotide phosphodiesterases (PDEs) are members of a superfamily of enzymes involved in regulating the intracellular levels of the second messengers cyclic nucleotide AMP (cAMP) and guanosine 3′5′-cyclic monophosphate (cGMP) [1]. Intracellular cyclic nucleotide levels increase in response to extracellular stimulation by hormones, neurotransmitters, or growth factors and are downregulated through hydrolysis catalyzed by phosphodiesterases (PDEs) [2,3]. Thus, PDEs regulate a myriad of physiological processes and are implicated in genetic diseases, as well as associated with pathophysiology of the nervous and cardiovascular system, fertility, autoimmune diseases, and cancer [1,2,3,4].

Human PDEs are derived from 21 genes separated into 11 families (*PDE1* to *PDE11*) and classified by amino acid sequences, regulatory properties, and catalytic characteristics, which are grouped by the homology of their conserved C-terminal catalytic domains [1,2,3]. The N-terminal portion of PDE molecules defines the specific properties of each member and variant of the PDE gene family [1,2,3]. Transcription from different initiation sites and differential splicing of their mRNAs results in multiple isoforms that are distinguished primarily by their substrate selectivity (cAMP versus cGMP) and modes of regulation. Each isoform is distinct due to its unique expression pattern at the level of the tissue or organ, cell type and subcellular compartment, and susceptibility to pharmacological inhibition, which has provided many possibilities for identifying increasingly selective therapeutic targets [3,4].

Cancer is driven by genetic and epigenetic changes, which lead to altered signaling pathways that control cell division, cell death, and cell motility, thereby fueling wider signaling networks that favor cancer progression. Regulation of cyclic nucleotide signaling is considered one of several components involved in biological processes, such as cell proliferation and energy homeostasis. Indeed, various alterations leading to activation or inactivation of key components of cAMP and cGMP signaling pathways occur in several pathophysiological conditions, including tumorigenesis [1]. Several studies have demonstrated that activation of cyclic nucleotide signaling through one of three mechanisms: Induction of cyclic nucleotide synthesis, inhibition of cyclic nucleotide degradation, or activation of cyclic nucleotide receptors is sufficient to inhibit proliferation and activate apoptosis in many types of cancer cells [5].

Many carcinomas and hematological malignancies have been associated with reduced levels of cAMP and/or cGMP secondary to an elevation in PDE activity [6,7,8,9,10]. Chronic lymphocytic leukemia cells exhibit increased *PDE7B* expression [11]; *PDE5* is strongly expressed in glioblastoma multiforme [12] and colon cancer [13], and glioma cells overexpress one or more isoforms of *PDE4* [14]. Furthermore, several PDE isoforms are present in granulosa cells as well as in oocytes in preovulatory follicles of the mammalian ovary regulating the meiotic cell cycle [15]. Additionally, many PDEs are expressed in cells of the spermatogenic pathway where they may regulate sperm motility [16], and *PDE5* is expressed in the contractile tissues of the male excurrent duct and accessory glands where its increased activity contributes to erectile dysfunction [16].

Various cellular and molecular alterations of the cAMP-signaling pathway have been observed in endocrine diseases. Studies show that *PDE2A*, *PDE8A*, *PDE8B*, and *PDE11A* are the major PDEs expressed in the adrenal cortex and play a role in adrenal physiology [17]. Aberrant cAMP signaling has been linked to genetic forms of cortisol excess which can lead to Cushing’s syndrome and related adrenal hyperplasia [17]. Variants in *PDE8B* predispose to primary pigmented nodular adrenocortical disease (PPNAD), a bilateral form of micronodular adrenal hyperplasia that causes ACTH (adrenocorticotropic hormone)-independent Cushing’s syndrome [18]. A higher frequency of missense variants of *PDE11A* has been found in adult patients with macronodular adrenocortical hyperplasia and adrenocortical tumors (ACTs) than in control patients [19].

The role of inactivating variants in PDEs in pediatric ACTs has not been investigated thoroughly—unlike in adrenocortical hyperplasia and in such tumors in adults. In the present investigation, we examined the frequency of germline and acquired PDEs variants in a cohort of pediatric patients. Our findings suggest the potential involvement of PDEs in pediatric adrenocortical tumorigenesis.

## 2. Results

### 2.1. Discovery Cohort of Pediatric ACT Patients Harboring PDE Variants

**Figure 1:**
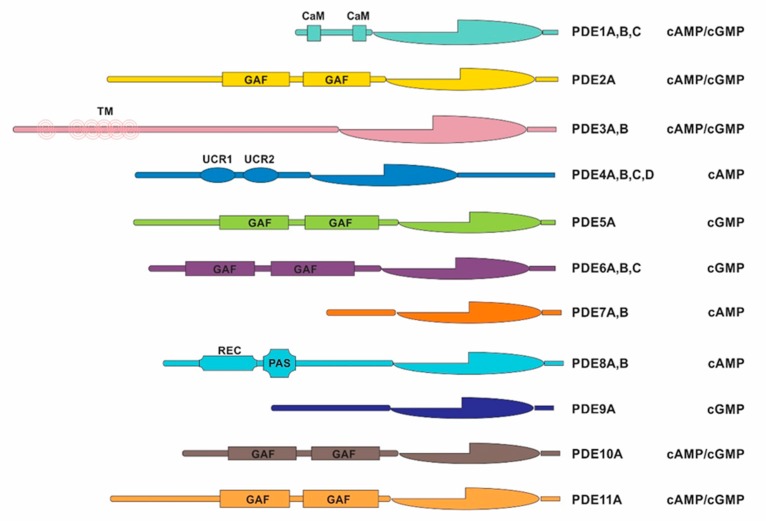
Schematic representation of human phosphodiesterase genes. Phosphodiesterases (PDEs) are organized into 11 families with specific adenosine 3′5′-cyclic monophosphate (cAMP) and/or guanosine 3′5′-cyclic monophosphate (cGMP) substrates (identified on the right). CaM, calmodulin-binding domain; GAF, cGMP-binding PDEs *Anabaena* sp. adenylyl cyclase and *Escherichia coli*. FhlA; TM, transmembrane domain; REC, signal receiver domain; PAS, Per-Arnt-Sim domain; UCR, upstream conserved region.

Whole genome sequencing (WGS) and whole exome sequencing (WES) data of 37 children with ACTs (“discovery cohort”) were retrieved for analyzing germline and acquired variants in PDE family genes (Figure 1) and other cAMP/cAMP-dependent kinase (PKA)-signaling pathway genes (*PDE4DIP*, *CREB*, *GNAS* and *PRKACA*). Demographics and clinical data for these patients are shown in Table 1.

### 2.2. PDE Variants Identified in the Discovery Cohort

**Figure 2:**
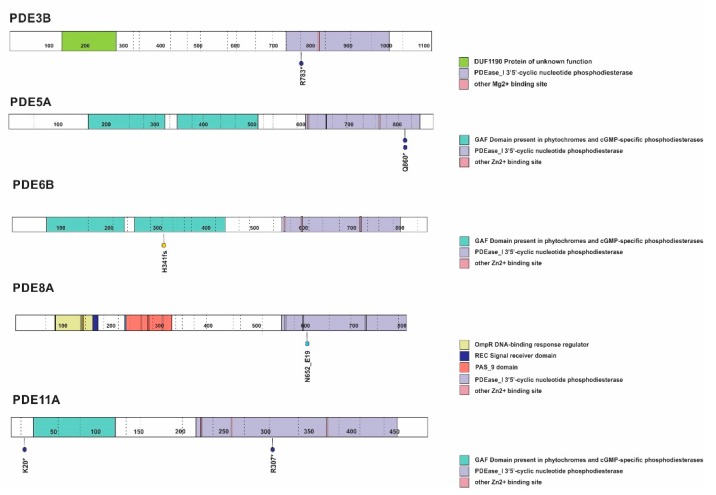
Phosphodiesterase variants in the discovery cohort. Germline inactivating variants in PDEs (dark blue, nonsense; light blue, splice-site; and yellow, frame-shift variants). Protein domains shown on right. Illustration based on PeCan Data Portal (https://pecan.stjude.cloud/home).

**Figure 3:**
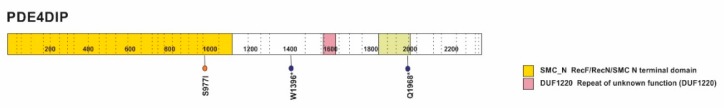
*PDE4DIP* variants identified in the discovery cohort. Germline and acquired inactivating variants in *PDE4DIP* (dark blue, nonsense; and orange, missense variants). Protein domains shown on right. Illustration based on PeCan Data Portal (https://pecan.stjude.cloud/home).

Sequencing analysis of genomic DNA from 37 pediatric ACT patients revealed the presence of germline-inactivating variants in PDEs and related genes in 9 (24%) patients (Figure 2 and Figure 3 and Table 1). Inactivating germline nonsense variants were documented in *PDE3B* (OMIM 602047, NM_000922.4) (p.R783*, c.2347C > T, rs150090666, gnomAD frequency, 0.06%), *PDE5A* (OMIM-603310, NM_001083.4) (2x p.Q860*, c.2578C > T, rs140289122, 0.17%) and *PDE11A* (OMIM-604961, NM_016953.4) (p.K20*, c.58A > T rs148183964, 0.06% and p.R307*, c.919C > T, rs76308115, 0.29%). Additional structural germline variants were observed in *PDE6B* (OMIM-180072, NM_000283.3 (p.H341Qfs*23) and *PDE8A* (OMIM-602972, NM_002605.3) (c.1953-4A > G splice region) (Figure 2). Excluding the p.K20* variant for *PDE11A* and p.H341Qfs*23 for *PDE6B*, all other variants were found in the PDE catalytic domain (Figure 2). Additional germline nonsense variants were verified in *PDE4DIP* (OMIM-608117, NM_001198834.3) (p.W1396*, c.4187G > A, rs782516582, 0.008% and p.Q1968*, c.5902C > T, new variant) (Figure 3). Notably, analysis of tumor DNA revealed loss of heterozygosity (LOH), with retention of the mutant allele in all cases. Additional rare germline variants maintained in tumor samples due to LOH and not reported in ClinVar (https://www.ncbi.nlm.nih.gov/clinvar/) were also observed and included in Table S1. Acquired alterations in PDEs and other cAMP/PKA signaling pathway genes were observed in three cases. The *GNAS* (OMIM-139320, NM_000516.6) (p.R201H, c.602G > A) pathogenic variant in addition to the *PDE4DIP* p.S977I missense variant was observed in the tumor sample from patient #3. The *GNAS* p.R201C, c.601C > T variant was observed in the tumor sample of patient #11, and a gene fusion [chr5:58476419(-)::chr2:212615429(-)] showing *PDE4D* exon 5 fused to *ERBB4* exon 5 was observed in the ACT from patient #10 (Table 1). No pathogenic or likely pathogenic variants were identified in *CREB* or *PRKACA* in this cohort.

Analysis of the TCGA whole exome sequence database representing 92 paired germline and adult ACC cases [20] revealed rare germline PDE variants that were retained in the tumor due to LOH and lacked representation of pathogenicity in Clinvar (Table S2). Of note, four somatic inactivating PDE variants (*PDE2A*, p.C935*; *PE3A*, p.E319*; *PDE8A*, c.1735-3C > A; and *PDE8A*, p.S386Hfs*4) were identified in this cohort. An acquired variant in *PDE4C* (p.A291G; COSV53206356) was also reported in an adrenocortical carcinoma among 41 adult cases analyzed by WES [21].

### 2.3. Transcriptome Profiling of PDEs in the Discovery Cohort

**Figure 4:**
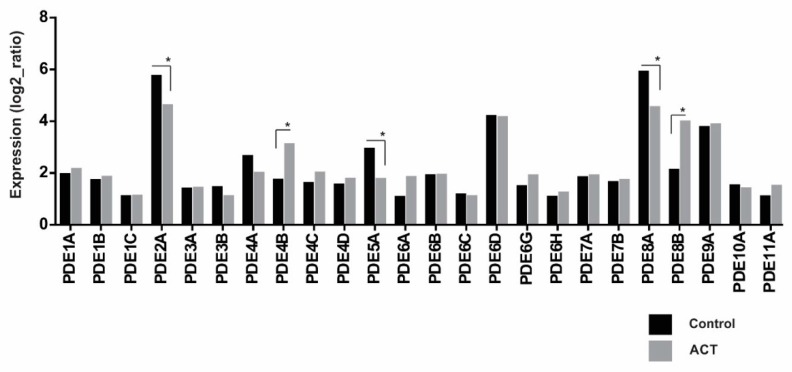
Transcriptome profiling of phosphodiesterase genes in pediatric adrenocortical tumors and normal adrenal. Significant overexpression of *PDE4B* and *PDE8B* and downregulation of *PDE2A*, *PDE5A,* and *PDE8A* were observed in adrenocortical tissues. (* *p < 0*.05; Bonferroni test—GraphPad Prism, v6, (GraphPad, San Diego, CA, USA)).

Transcriptome profiling of pediatric ACTs (*n* = 16) and normal adrenal cortex samples (*n* = 6) revealed that *PDE2A*, *PDE6D*, *PDE8A*, and *PDE9A* are highly expressed in both normal and adrenal tumor tissue, compared to other PDE family members. No significant differences in expression were observed when comparing tumor tissue and normal adrenal for most PDE genes. However, significant overexpression of *PDE4B* and *PDE8B* and downregulation of *PDE2A*, *PDE5A*, and *PDE8A* were observed in pediatric ACTs compared to normal adrenal (Figure 4).

### 2.4. Germline PDE Variants Identified in Pediatric ACTs Associated with the Founder TP53 p.R337H Variant

Whole exome analysis of germline DNA from an independent cohort of 18 pediatric ACT patients harboring the *TP53* p.R337H allele revealed additional inactivating variants in *PDE6A* (p.K827del) and *PDE11A* (p.K119Sfs*2). Nonsense variants were also observed in *PDE4DIP* (p.E515*, c.1543G > T, new variant / p.E1745*, c.5233G > T, new variant). Additional rare germline variants with no representation of clinical significance in ClinVar (https://www.ncbi.nlm.nih.gov/clinvar/) observed in this cohort are included in Table S3.

## 3. Discussion

In this study, we identified nine germline inactivating variants in phosphodiesterases and related genes in a discovery cohort of 37 pediatric patients with ACT (24%). Inactivating variants were observed in *PDE3B*, *PDE5A*, *PDE6B*, *PDE8A*, and *PDE11,* and the phosphodiesterase interacting protein *PDE4DIP*. All observed variants, except for two, were in the catalytic domain, which suggests a loss of function of PDE [22]. Of these, only *PDE11A* p.R307* has been previously found in association with Cushing’s syndrome due to micronodular adrenocortical hyperplasia in a female carrier [19,23]. In our study, the female patient harboring the *PDE11A* p.R307* variant (Patient #3) developed an aldosterone-producing tumor at the age of 12 years. In addition to the germline *PDE11A* nonsense variant, the tumor acquired a pathogenic variant in *GNAS.* These findings agree with the role of *PDE11A* in genetic predisposition to the development of adrenal tumors [19,23], and that additional cooperating events leading to altered cAMP/PKA signaling [24] are required to drive adrenocortical tumorigenesis.

The most widely-used and valuable histopathological scoring criteria for predicting pediatric adrenocortical tumor malignancy is the Wieneke classification system [25], which relies on nine macroscopic and microscopic variables. Based on this system, tumors are classified as adenoma (ACA; 0–2 variables), undetermined malignant potential (Und; 3 variables), and carcinoma (ACC; 4 or more variables), which portends a poor clinical outcome [25]. However, lack of definitive and reliable histopathological criteria for malignancy is still a challenge for pediatric ACT. About 50% of pediatric ACTs are associated with germline *TP53* variants that lead to more complex genomic landscapes [26]. Although discrete genomic changes were not independently associated with prognosis, complex genomic alterations tended to portend an unfavorable outcome [27]. Furthermore, *TP53* variants observed in pediatric adrenocortical tumors did not correspond to the conventional hotspot variants associated with classic Li–Fraumeni syndrome (LFS), and most retain a wide range of functionality [28,29]. Consistent with these observations, among the five carriers of *TP53* variants in our cohorts, only one (patient #4) harbored a predicted nonfunctional *TP53* variant [28], and none were classified as LFS. 

Loss of heterozygosity was observed for all inactivating phosphodiesterase/phosphodiesterase-related variants. One can hypothesize that a complete inactivation of those proteins, due to an inactivating variant in one allele and the loss of the second wild-type allele, in the right environment could favor the development of tumors. The “right” environment could be formed by the presence of a *TP53* hypomorphic variant, that predisposes the cells to form tumors. A previous study demonstrated that PDEs can act as phenotype modifiers, leading to adrenal tumors in Carney complex patients carrying *PRKAR1A* variant [30].

A high frequency of PDE variants was observed in patients with prostate cancer [31]. The pCREB:CREB ratio (phosphorylated cAMP response element-binding protein: cAMP response element-binding protein) showed an imbalance in the cAMP availability, probably due to downregulation of some of the PDE molecules [31]. In the present work, when the transcriptome analysis was accessed, a decrease of *PDE2A*, *PDE5A*, and *PDE8A* expression and a significant overexpression of *PDE4B* and *PDE8B* was observed in adrenocortical tumors, compared to normal adrenal tissue. PDEs have been found to play critical roles in modulating multiple signaling pathways. The presence of consistent and significative differences in the expression of PDEs throughout patients’ tumors as compared with that in controls could indicate an imbalance of the cAMP pathway. Corroborating this hypothesis, a knockout mouse for the *Prkar1a* gene in the pancreas leads to neuroendocrine tumorigenesis, probably due to the dysregulation of the (cAMP)-dependent kinase (PKA) pathway [32]. 

Of interest, we have documented germline nonsense variants in *PDE4DIP*, a protein that interacts with the cyclic nucleotide phosphodiesterase *PDE4D* to anchor this protein to the Golgi/centrosome region of the cell. This gene has been associated with myeloproliferative disorders, as shown by its fusions with platelet-derived growth factor receptor beta gene (*PDGFRB*) [33]. In addition, *PDE4DIP* variants are one of the most frequent in metastatic adrenocortical carcinomas in adults [34]. Of note, all four of our patients with an inactivating variant in *PDE4DIP* also harbor the hypomorphic widespread *TP53* p.R337H founder allele [35,36,37]. These findings suggest that *PDE4DIP* variants (and perhaps others c-AMP signaling pathway genes) predispose *TP53* p.R337H carriers to adrenocortical tumorigenesis.

Genome-wide associations studies demonstrate that *PDE2A*, *PDE8B,* and *PDE11A* modulate steroidogenesis [17] and are associated with adrenal Cushing’s syndrome and/or bilateral adrenal hyperplasia [17]. In contrast, our findings identified germline inactivating PDE variants in patients who developed adrenocortical carcinomas, including cases with virilization, and aldosterone-producing tumors. The observed concomitant PDE variants and hypomorphic *TP53* alleles or chromosome 11p uniparental disomy in the germline of pediatric ACT cases support the combined additive effect of multiple genetic variants in cancer susceptibility [38].

In this study, we added PDE variants as a candidate causative gene for pediatric adrenocortical lesions. Altogether, sequencing analysis and transcriptome profiling support the importance of alterations in the cAMP signaling pathway in adrenocortical tumors. As we learn more about the functional roles and molecular interactions of each PDE, as well as how the variants operate in adrenocortical tumorigenesis, we will better understand the full potential of PDEs as therapeutic targets.

## 4. Materials and Methods

### 4.1. Phosphodiesterase Variants in the Discovery Cohort

We retrieved data from primary ACTs and matched peripheral blood DNA analyzed by WGS (*n* = 19) at an average 41.9x coverage or by WES (*n* = 18) at an average 84.8x coverage. Of the nine cases with germline variants in PDEs, six were disclosed by WGS and three by WES [26]. WGS and WES were performed as previously described [26]. Sequencing data for variants in the PDE family of genes (*PDE1* to *PDE11*, Figure 1), and *PDE4DIP* as well as cAMP/PKA-signaling pathway genes *GNAS*, *PRKACA*, and *CREB* were analyzed. Detected germline variants were evaluated in tumor tissue to determine heterozygosity status.

### 4.2. Transcriptome Profiling

Transcriptome profiling was performed by using total RNA extracted from pediatric ACTs (*n* = 16) in the discovery cohort. Six samples of normal adrenocortical tissue obtained during nephrectomy for Wilms tumor were used as controls in gene expression studies. Library construction and sequencing was performed as previously described [26]. The RNA expression level of PDE genes was measured as fragments per kilobase of transcript per million fragments mapped [26].

Sequencing and transcriptome data were retrieved from the European Genome-phenome Archive (EGA) under accession code EGAS00001000192.

### 4.3. Whole Exome Sequencing of an Independent Cohort of Pediatric ACTs Harboring the Germline TP53 p.R337H Variant

Peripheral blood DNA was isolated from 18 children with ACT and 36 cancer-free parents and analyzed by WES. All patients with ACTs tested positive for the germline *TP53* p.R337H variant. Written informed consent was obtained from all participants. This research was approved by the Pequeno Príncipe Hospital Ethics Committees (Curitiba, PR, Brazil, under ethic codes CAA: 0023.0.208.000-05 (2005), and CAAE 0612.0.015.000-08 (2009 and 2012).

Genomic DNA was isolated from blood samples using the ReliaPrep™ Blood gDNA Miniprep System (Promega, Madison, WI, USA) and quantified with the Qubit® 3.0 Fluorometer and Qubit dsDNA HS assay kit (Thermo Fisher Scientific, Grand Island, NY, USA). WES was performed by using the SureSelect Human All Exon V5 (Agilent Technologies, Santa Clara, CA, USA) for sequence capture and Illumina HiSeq2500 (2 × 125 bp paired-end) for sequencing (Illumina, San Diego, CA, USA). Read alignment, variant calling, prioritization, and filtering were performed on the Sirius online platform (Integragen, Evry, France). A custom Python script was used to compute filtered variant data into tables.

## 5. Conclusions

We reported recurrent inactivating germline PDE variants in association with pediatric adrenocortical tumors. In each case, the wild-type allele was selected against by LOH, suggesting an imbalance of the cAMP signaling pathway contributes to tumor progression.

## Figures and Tables

**Table 1:** Clinical data of pediatric adrenocortical tumor (ACT) patients with germline and acquired PDE and cAMP-signaling genes variants.

Case	c-AMP Pathway/ Germline	c-AMP Pathway/ Somatic	*TP53* status	Gender	Clinical Presentation	Histology	Age at diagnosis (months)	Tumor weight (g)	Stage	Survival Status
1	p.Q860*-*PDE5A*		WT	F	V	ACA	59.8	20.5	I	Alive
2	p.Q860*-*PDE5A*		p.R337H	F	V	ACC	38.0	6	I	Alive
3	p.R307*-*PDE11A*	p.S977I-PDE4DIP/p.R201H-GNAS	WT/UPD	F	A	Und	140.6	388	III	Alive
4	p.K20*-*PDE11A*		p.T125T	M	V	ACC	103.0	500	III	Alive
5	p.R783*-*PDE3B*		WT	F	V	Und	26.2	69	I	Alive
6	p.W1396*-*PDE4DIP*		p.R337H	M	V	ACC	21.0	Unk	I	Unk
7	p.Q1968*-*PDE4DIP*		p.R337H	M	V+C	ACC	24.4	Unk	I	Died
8	p.H341Qfs*23-PDE6B		p.R337H	F	V	ACC	35.0	30	I	Alive
9	c.1953-4A>G -PDE8A		WT/UPD	F	C	ACA	17.0	120	III	Alive
10		PDE4-ERRB4	p.R273C	F	NF	ACC	24.0	Unk	IV	Died
11		p.R201C-GNAS	WT	F	V	Und	83.0	255.7	II	Alive

ACA, adrenocortical adenoma; ACC, adrenocortical carcinoma; Und, Undetermined, WT, wild-type; F, Female; M, Male; V, virilization; A, aldosterone producing tumor; C, Cushing; NF, non-functional; R, right; L, left; Unk, Unknown.

## Supplementary Materials

The following are available online at https://www.mdpi.com/2072-6694/12/2/506/s1, Table S1: Additional variants observed in the discovery cohort of pediatric adrenocortical tumors, Table S2: Acquired and constitutive PDEs variants in TCGA database, Table S3: Additional variants observed in an independent cohort of carriers of the germline TP53 p.R337H variant.
